# Spatial inequalities in skilled attendance at birth in Ghana: a multilevel analysis integrating health facility databases with household survey data

**DOI:** 10.1111/tmi.13460

**Published:** 2020-07-18

**Authors:** Winfred Dotse-Gborgbortsi, Andrew J. Tatem, Victor Alegana, C. Edson Utazi, Corrine Warren Ruktanonchai, Jim Wright

**Affiliations:** 1 School of Geography and Environmental Science, University of Southampton, Southampton, UK; 2 WorldPop Research Group, School of Geography and Environmental Science, University of Southampton, Southampton, UK; 3 Population Health Unit, Kenya Medical Research Institute – Wellcome Trust Research Programme, Nairobi, Kenya; 4 Faculty of Science and Technology, Lancaster University, Lancaster, UK; 5 Southampton Statistical Sciences Research Institute, University of Southampton, Southampton, UK

**Keywords:** skilled birth attendance, maternal health, GIS, travel time, quality care, EmONC, SDG 3 (good health and well-being), SDG 5 (gender equity), SDG 10 (reduced inequalities), SDG 11 (sustainable cities and communities), SDG 16 (peace, justice and strong institutions), SDG 17 (partnerships for the goals)

## Abstract

**Objective:**

This study aimed at using survey data to predict skilled attendance at birth (SBA) across Ghana from healthcare quality and health facility accessibility.

**Methods:**

Through a cross-sectional, observational study, we used a random intercept mixed effects multilevel logistic modelling approach to estimate the odds of having SBA and then applied model estimates to spatial layers to assess the probability of SBA at high-spatial resolution across Ghana. We combined data from the Demographic and Health Survey (DHS), routine birth registers, a service provision assessment of emergency obstetric care services, gridded population estimates and modelled travel time to health facilities.

**Results:**

Within an hour’s travel, 97.1% of women sampled in the DHS could access any health facility, 96.6% could reach a facility providing birthing services, and 86.2% could reach a secondary hospital. After controlling for characteristics of individual women, living in an urban area and close proximity to a health facility with high-quality services were significant positive determinants of SBA uptake. The estimated variance suggests significant effects of cluster and region on SBA as 7.1% of the residual variation in the propensity to use SBA is attributed to unobserved regional characteristics and 16.5% between clusters within regions.

**Conclusion:**

Given the expansion of primary care facilities in Ghana, this study suggests that higher quality healthcare services, as opposed to closer proximity of facilities to women, is needed to widen SBA uptake and improve maternal health.

## Introduction

Skilled attendance at birth (SBA) is a proven intervention to reduce avoidable deaths related to pregnancy and childbirth [1]. Thus, high maternal mortality in Ghana and other sub-Saharan African countries is partly attributed to low uptake of SBA. Between 1990 and 2015, investments have led to reductions in maternal mortality but sub-Saharan Africa continues to report more than 500 maternal deaths per 100 000 live births, the highest prevalence of any sub-region globally [2]. Besides, high maternal mortality in health facility births indicates the low quality of emergency obstetric care in some settings [3].

Geographical factors such as rurality, proximity to a health facility providing quality services, road networks, water bodies and elevation are important determinants of SBA [4–6]. When a woman is in labour and needs emergency care, geographical barriers such as bridges in disrepair, poor roads and prompt access to and cost of reliable transport can limit their timely access to the nearest well-equipped health facility providing SBA [7, 8].

Studies have identified individual and geographical factors that influence the use of SBA services. Some of the important individual-level determinants are education, wealth, age, health insurance, parity and quality of antenatal care received [9–12]. Women’s ability to afford maternal health services, understand risks of childbirth and availability of comprehensive maternal health services are important in choosing where to give birth.

Maternal health studies applying geospatial methods reveal inequalities in access to services. Some studies calculate the service to demand ratio within districts, ignoring possible cross-border movement [13]. An enhanced analysis modelled travel time to health facilities and measured the number of women with access to emergency obstetric and newborn care (EmONC) within different travel time bands [14] but did not relate their results to the use of SBA. Another study used geographically weighted regression to predict SBA using Demographic and Health Survey data (DHS) but only mapped predictions at cluster locations [15]. In five East African countries, a multilevel model was used to produce high spatial resolution probabilities of SBA without accounting for the quality of care [6]. To date, no study has assessed the influence of travel time and the quality of care on SBA in a multilevel model while accounting for individual determinants and predicted SBA at high spatial resolution.

Therefore, this study’s objective was to examine the effect of proximity and the quality of care on SBA by integrating routine health management information systems (HMIS), survey and service provision assessment (SPA) data to model SBA. As a secondary objective, we assessed if HMIS data improved SBA predictions. We aggregate travel time and probability maps to policy-relevant administrative units to inform planning, distribution of resources and targeted efforts to reduce inequalities. Through a cross-sectional observational study design, we used a random intercept mixed effects multilevel logistic modelling approach to estimate the odds of having SBA and then applied model estimates to spatial layers to assess the probability of SBA at high spatial resolution across Ghana.

## Methods

### Data sources

We used (A) births, GPS and demographic data from the 2017 DHS Ghana Maternal Health Survey (GMHS) [16]; (B) location and count of births in health facilities in 2017 from the Ghana Health Service (GHS) HMIS; (C) gridded estimated births data from the WorldPop project to weight probabilities [17, 18]; (D) SPA data on emergency obstetric and newborn care in health facilities to quantify healthcare quality near DHS cluster locations [19]; (E) urban settlement extents for predicting SBA at high spatial resolution [20]; and (F) spatial data (roads, rivers, elevation, and land cover) for modelling travel time to health facilities [21–23]. Clusters are sampled enumeration areas where women are interviewed. The DHS collects longitude/latitude information from clusters but deliberately displaces them 5 km in rural or 2 km in urban settings for privacy and confidentiality reasons [24].

The GMHS sampled 22 062 women aged 15–45 years, equally distributed between rural and urban clusters, who were interviewed between June and October 2017. After excluding women who had never given birth, women who did not respond to the SBA question and women from three clusters without location data, 11 656 women were included in the final analysis (Supplementary Material, Figure S1). Of 4470 health facilities reporting to GHS, 2417 were providing birthing services.

### EmONC survey and quality care

Quality of care near clusters was assessed using the 2010 EmONC SPA nationwide census data for 1268 health facilities between July and September 2010. The census included all health facilities averaging five births per month in 2009 but used a lower number of births as facility inclusion criteria for the Northern, Upper East and Upper West regions due to lower coverage of health facility births in those regions. Details of the EmONC survey methods and classification of EmONC status can be found here [19]. The EmONC classes (comprehensive, basic, partial and non-EmONC) were defined based on the number of essential obstetric care signal functions performed in a health facility according to WHO recommendations [25]. There are nine signal functions, basic facilities provide seven functions, and comprehensive facilities provide the five basics plus caesarean and blood transfusion. Partial facilities have one or two signal functions missing from their expected services, and non-EmONC facilities did not meet the partial, comprehensive or basic criteria [19, 25].

### Travel time to health facilities

Two travel modes, walking and motorised, were implemented in AccessMod 5.0 [26, 27] to model walking from a DHS cluster point to the nearest road and continuing the journey with a motorised vehicle to a health facility [28]. Travel times were modelled from the DHS cluster location to (i) the nearest health facility, (ii) the nearest health facility providing birthing services and (iii) the nearest hospital. We modelled geographic accessibility using land cover, roads, a digital elevation model and water bodies. We applied 5 km/h in grassland, low vegetation, shrubs, woodlands, forests, croplands; 5 km/h within cities; and 3 km/h walking speed for non-mechanised travel based on modified estimates from Alegana *et al*. [28]. Mechanised network travel speed on roads was based on maximum speed limits (primary = 90 km/ h, secondary = 50 km/h, tertiary = 30 km/h) specified in Ghana’s 2004 Road Traffic Law (2004 Road Traffic Act 683). Tobler’s function was applied to account for the variability in walking speed when climbing or descending slopes [29]. Water bodies were only traversable when crossed by roads. See Supplementary Table S1 for travel speeds assigned to land cover types and mechanised/non-mechanised travel.

The modelled travel times were extracted for GMHS using cluster locations. We extracted the median travel time within 5-km and 2-km buffers for rural and urban clusters, respectively. The buffers account for the deliberate displacement/distortion of cluster locations to protect the confidentiality of respondents [24].

### Multilevel modelling

A three-level random intercept mixed effects generalised linear model was used to model the probability of having SBA. A multilevel model approach was adopted due to the nested complex survey design in the DHS, in which women are nested in clusters and clusters nested in regions. Furthermore, the multilevel model accounts for contextual cluster-level factors otherwise not captured as variables in the survey [30]. The individual fixed effects were age, education, health insurance, antenatal care, parity and wealth. The cluster or community fixed effects were rurality, quality of maternal care near cluster (EmONC status) and travel time to the nearest health facility, with random effects modelled for clusters and regions. These variables were chosen based on their importance in the literature as determinants of SBA [6, 7, 9].

We used a generalised linear model with a binomial logit link function to predict SBA because of the variable’s binary nature, indicating whether a woman had SBA or not. A woman was classified as having SBA if she reported that a midwife, doctor or nurse assisted their most recent birth. If two or more persons were present, the most competent individual was used to determine the classification.

The DHS wealth categories (richest, rich, middle, poor and poorest) were simplified to three (rich, middle and poor) by collapsing the top and bottom two categories. The DHS estimated the wealth index by principal component analysis using basic consumer items (e.g. television and bicycle/car) and housing characteristics (e.g. drinking water, toilet, flooring materials) [16].

The multilevel models were implemented in R using the glmer function from the lme4 package [31]. Initially, we compared a logistic regression model with multilevel null models using a likelihood ratio test to determine the improvement in model fit from using a multilevel model. We compared the models using the Akaike information criterion and checked for multicollinearity using the generalised variance inflation factor with ten as the threshold. Also, we calculated variance partition coefficients (VPC) to estimate the unobserved effects of cluster and region on SBA. We weighted frequencies and proportions to ensure representative estimates because of the complex survey design.

### High-resolution probability surfaces

To derive the 100 × 100 m probability maps, we stacked the travel time surface, EmONC layer (2 km buffer) and the urban extent [20] and multiplied them with the model coefficients (Equation 1). We defined a reference woman for easier interpretation. We characterised the reference woman as a poor woman with primary education; registered with health insurance; who attended four or more antenatal care (ANC) appointments; and has three or four children. Therefore, the individual characteristics of a woman were similar across space while travel time, rural/urban, and EmONC status varied. Also, we weighted the probability raster using the 2019 World-Pop estimated births for Ghana to derive estimates that account for events such as abortion and miscarriage [6]. Policy-relevant mean probabilities were summarised at district level (admin 2). The resulting map shows the probability of the reference woman having a skilled attendant at birth.

(1) $$\pi_{ijk}=\frac{\exp\left(\beta_{0}+\beta_{1}x_{1ijk}+\beta_{2}x_{2ijk}+\cdots +\beta_{n}x_{nijk}+v_{k}+u_{jk}+e_{ijk}\right)}{1+\exp\left(\beta_{0}+\beta_{1}x_{1ijk}+\beta_{2}x_{2ijk}+\cdots +\beta_{n}x_{nijk}+v_{k}+u_{jk}+e_{ijk}\right)}$$

### Ethics

We received approval from the DHS to use their data. The DHS sought informed consent from all participants in the survey. The University of Southampton granted ethical approval for secondary data analysis (Reference: 50661 Date: 3/7/2019).

## Results

### Travel time

**Figure 1:**
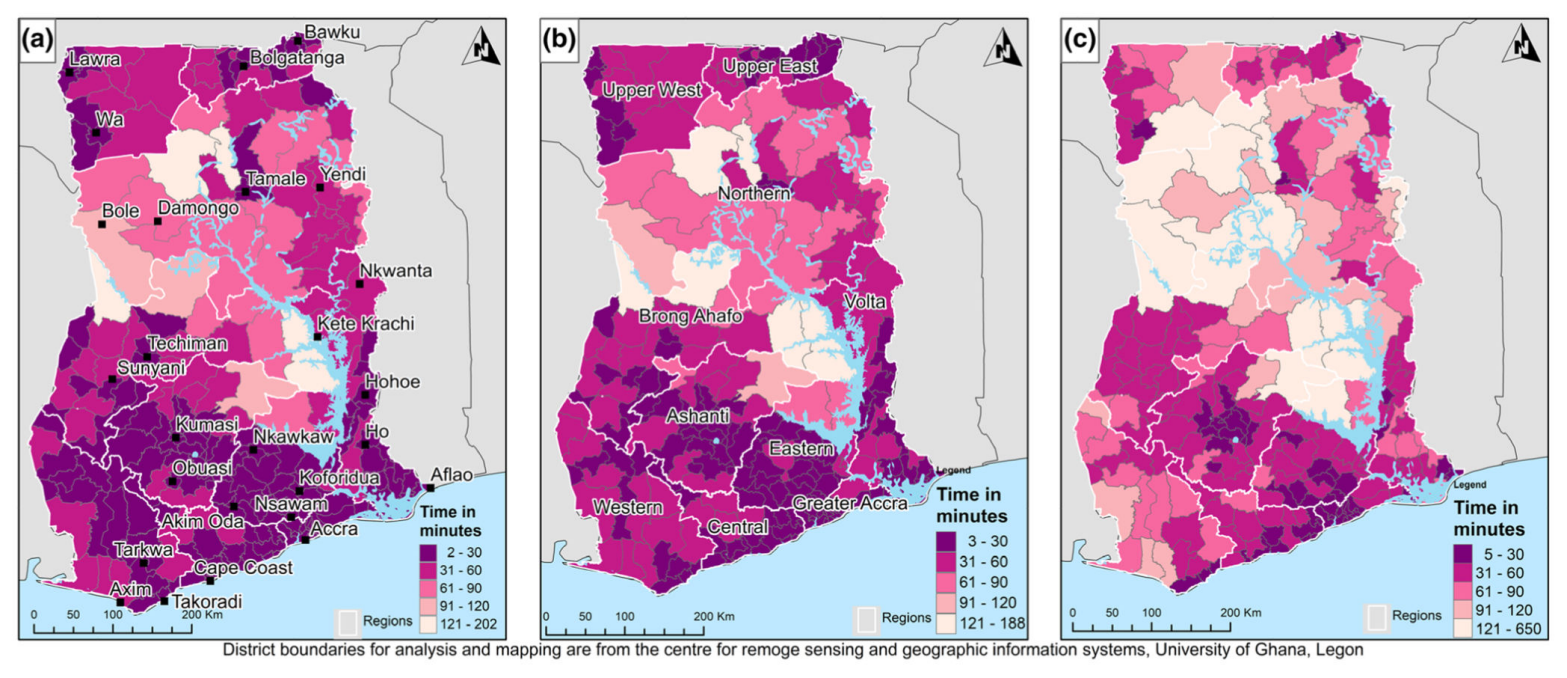
Median travel time for women residing in each district to the (a) nearest health facility, (b) nearest health facility providing birthing services and (c) nearest hospital providing birthing services [Colour figure can be viewed at wileyonlinelibrary.com]

Average travel times from the survey clusters were greater to hospitals (38 min) than to the nearest health facility providing birthing services (21 min) or to the nearest healthy facility (20 min). Figure 1 shows the median travel time to health facilities for women living in each district. Generally, it takes less than an hour to reach the nearest health facility. In Southern Ghana, most of the women were within 30 min drive time. Fewer women were within 30 min drive time to the nearest health facility providing birthing services compared with all health facilities. For hospitals providing birthing services, most women were within an hour’s travel time. The unaggregated travel time results are included as Supplementary Material, Figure S2.

### Sample characteristics and bivariate analysis

Table 1 shows the bivariate association between SBA and women’s sociodemographic characteristics. Most women had primary or no formal education, registered with health insurance, were between 20 and 30 years old, attended ANC four or more times, had 3 or 4 children, were poor and lived within an hour’s travel to a health facility. The proportion of women with SBA increased with higher education and decreased with higher birth order. A lower proportion of women used SBA if they did not have health insurance, lived in a rural area, attended ANC less than four times and were poor. There was no difference in SBA among different age groups (*P* = 0.265); however, all other variables were strongly associated (*P* < 0.001).

### Multilevel model

A likelihood ratio test showed that the multilevel null models with cluster and region random effects were significantly different from the null logistic regression model (*P* < 0.001). The goodness of fit of the three models (whether using travel time to any health facility, facilities providing birthing services or hospitals) differed significantly (*P* < 0.001), but the estimated effects were very similar. Therefore, we describe subsequent results in this section using travel to health facilities providing birthing services. As shown in Table 2, higher education, health insurance, attending more ANC appointments, wealthier households and living in an urban area were positive determinants of SBA. As the quality of maternal care, measured as EmONC status, increased near a cluster, the odds of SBA increased markedly. Compared with clusters that had no health facility nearby, health facilities not prepared for emergency obstetric care (non-EmONC) increased the odds of SBA by 38% and well-equipped facilities providing all essential care (Comprehensive EmONC) increased the odds of SBA 2.28 times. Each hour’s increase in travel time to the nearest health facility providing birthing services decreased the odds of SBA by 15%, but this effect was insignificant.

The estimated VPC suggests 7.1% of the residual variation in the propensity to use SBA is attributed to unobserved regional characteristics. Also, 16.5% lies between clusters within regions, whereas 76.3% lies between women within clusters. Compared with the null multilevel model, both cluster (chi-square: 1266.4, *P* < 0.001) and regional (chi-square: 119.85, *P* < 0.001) effects profoundly affected SBA. Variance of the random effects is presented in Table 3.

**Figure 2:**
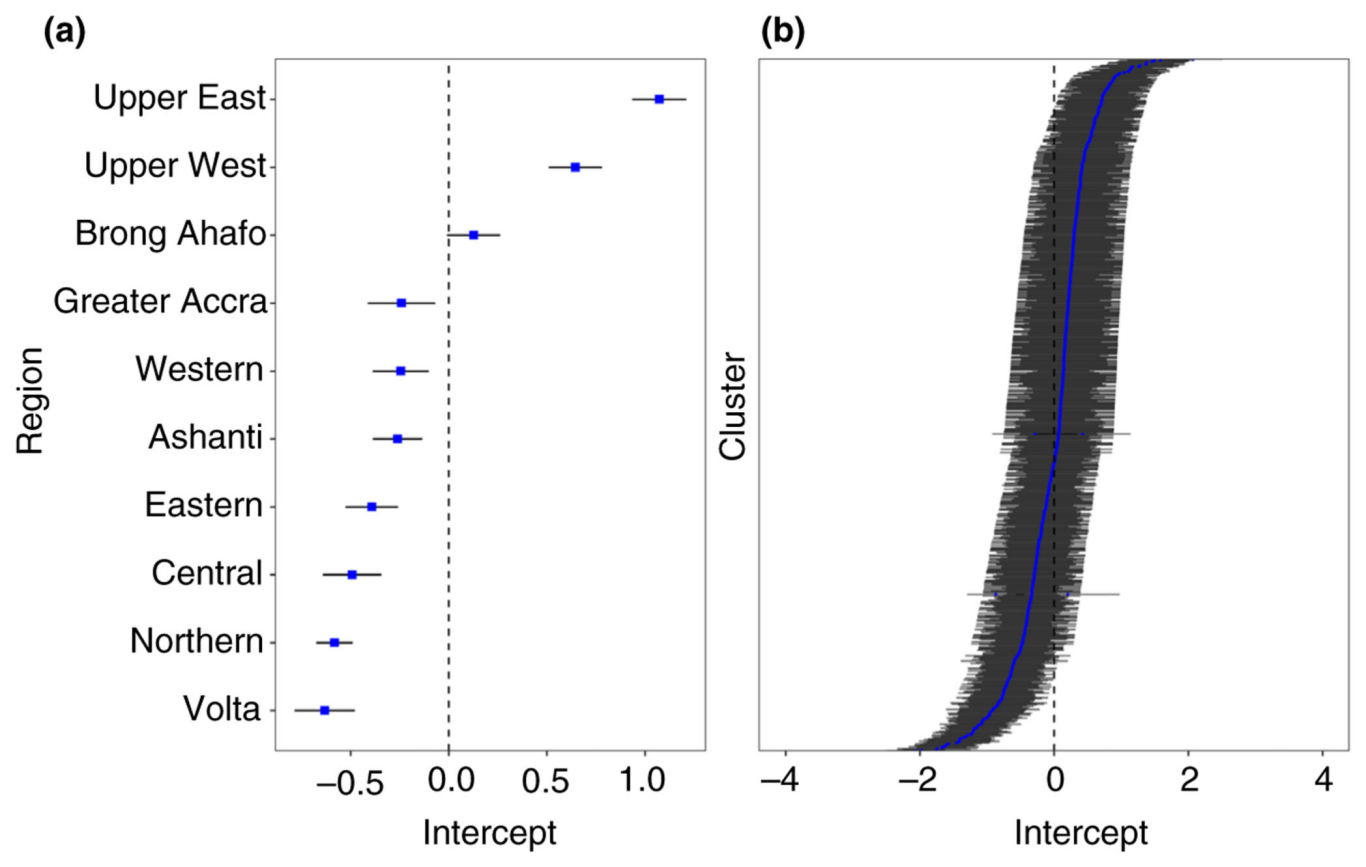
Caterpillar plots showing residuals from the multilevel model predicting skilled birth attendance in Ghana for (a) regions and (b) clusters, blue points are conditional variance from the multilevel model random effects and line segments showing standard deviation [Colour figure can be viewed at wileyonlinelibrary.com]

Model residuals (Figure 2a) showed the effect of region on SBA was highest in Upper East and lowest in Volta region. The cluster variance in Figure 2b shows few clusters were significantly above or below the average as most of the confidence limits crossed zero. Also, 60.8% of urban clusters were above the average SBA, 7% higher than rural locations. The confidence intervals for the clusters were similar and wide due to similar and small sample sizes within clusters.

### High-resolution predictions

**Figure 3:**
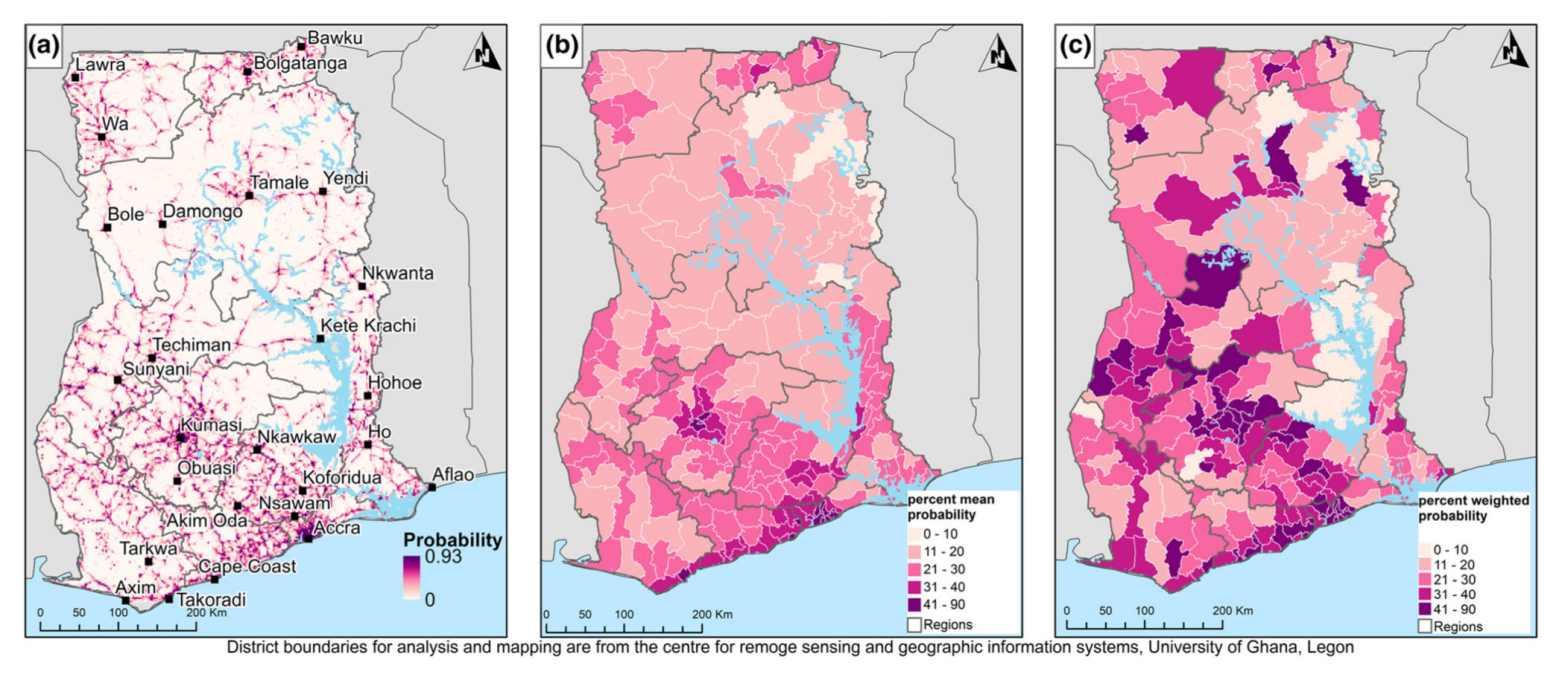
Probability of SBA by district at (a) 100 × 100 m resolution, (b) the district level (unweighted aggregation), (c) the district level (population-weighted aggregation) [Colour figure can be viewed at wileyonlinelibrary.com]

**Figure 4:**
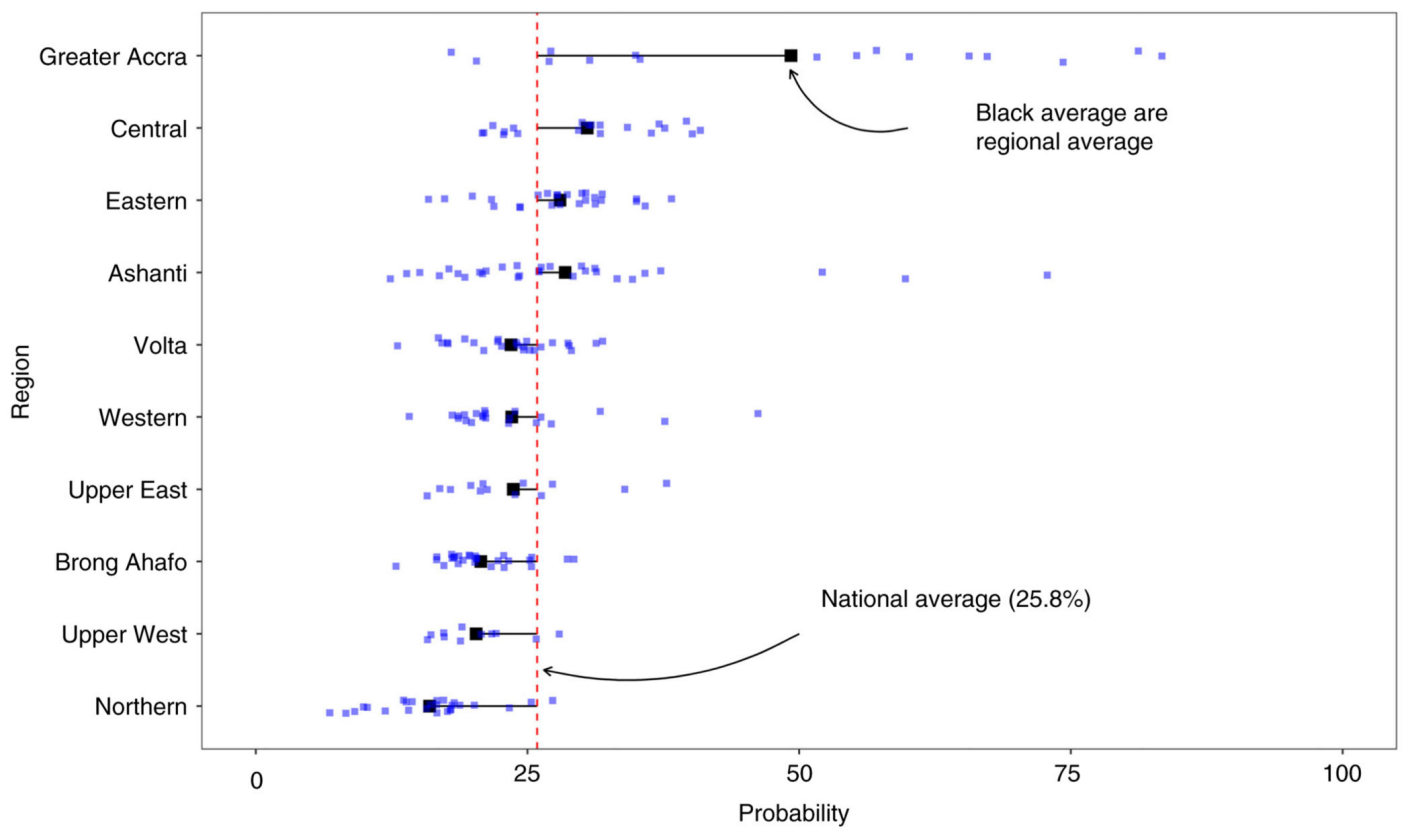
National (red line), regional (black squares) and district (blue points) average predicted probabilities of SBA [Colour figure can be viewed at wileyonlinelibrary.com]

The estimated probabilities are for the reference woman described in the methods. Generally, the southern parts of Ghana had higher probabilities of SBA compared with northern Ghana (Figure 3). Particularly, the estimated probability for most districts in Northern Region was less than 20%. Ashanti, Eastern and Greater Accra regions had the highest probabilities of SBA, mostly above 40%. When district prevalence of SBA was weighted using the estimated live births, the prevalence decreased in some districts. In Figure 4, the average probability for four regions (Greater Accra, Eastern, Central and Ashanti) was above the national average (25.8%) as most districts in the other regions were below average. Only two districts in Greater Accra were below the national average compared to the Northern, Brong Ahafo and Upper West regions where almost all districts were below it. There were few high probability outlier districts in Ashanti, Western and Upper East regions.

## Discussion

This analysis suggests higher EmONC status, indicating higher quality of SBA in health facilities increases uptake of SBA, as opposed to living closer to any health facility. Also, our findings on individual determinants of SBA such as rurality, education, parity, financial access and attending ANC appointments were similar to other studies (Table 2) [6, 9].

The travel time to health facilities did not significantly predict SBA. Although a study in Ghana has found that increasing travel time to a health facility significantly reduces SBA [4], our results are in line with others who found an insignificant relationship [32–34]. Furthermore, systematic review findings suggest that although increasing proximity to an obstetric facility generally reduces the probability of health facility births, some studies found an insignificant association [35]. The non-significant association might be due to the high density of health facilities in some parts of Ghana as Community Health Planning and Services (CHPS – the lowest level of primary care in Ghana) facilities with buildings have increased rapidly from 178 to 2017 within ten years (2006 to 2016) [36, 37]. Further analysis revealed that the average distance between health facilities was 4km and the spatial pattern of healthcare provision is significantly clustered. Therefore, women do not have to travel too far to reach the nearest health facility but higher quality is currently not universal. Higher service quality at health facilities near to DHS clusters positively influenced SBA.

Skilled attendance at birth has increased from 54 to 79 per cent within ten years (2007 to 2017) [16]. These percentages do not highlight inequalities within the women. The percentages reported in surveys are higher compared to our high-resolution estimates (Figure 3) because we estimated probabilities for a reference woman. We characterised this woman as being poor with primary education; registered with health insurance; having attended four or more antenatal care (ANC) appointments; having three or four children; and near a partial EmONC health facility. Evidently, the reference woman has lower chances of SBA (Table 2).

The impact of the free maternal health policy in Ghana reflects on the proportion of women with SBA and its uptake. Free maternal care and the minimal fee for health insurance has reduced some of the financial costs associated with childbirth in health facilities [38, 39]. Although free maternal care policy moderates the effect of wealth on SBA, women from wealthier households were more likely to have SBA. Similar policies such as increasing access to secondary education through the free senior high school policy are likely to implicitly reduce fertility and promote the uptake of SBA and impact maternal mortality.

Availability of data on the location of health facilities [40] and their quality of care are essential to improve service provision. The integration of different data streams demonstrates the potential of replicating such analysis in other countries and modelling additional maternal health indicators such as antenatal and postnatal care to improve previous studies [6]. Knowing the location of health facilities providing birthing services did not increase the predictive power of our model but including SPA data on the characteristics of health facilities revealed the importance of quality. Therefore, HMIS data containing fine spatial and individual details could help understand the unexplained geographic variations. Hence, there should be more investment in regularly collecting routine data on births as well as the availability, readiness and quality of service provision. Furthermore, SPA should collect more data on qualities of health facilities that attract women as well as clinical signal functions [41]. As geographic accessibility is reducing due to the increasing number of health facilities in some countries, frequent quality measurements are important to ensure every woman has access to the best maternal care at reasonable proximity.

Our study methods were informed by a review of studies investigating travel time and SBA [42]. In our study, drawing on review recommendations, we clearly defined the start and end of the journey as the cluster location and the nearest health facilities, respectively. Also, we explained that we used a travel time model that estimates travel using both walking and mechanised network measures in AccessMod. Therefore, we avoided recall bias errors related to using self-reported distance. Furthermore, our travel time method represents an improvement over straight line distance measures that do not consider road networks, topology or use of mechanised forms of transport.

### Limitations

Although we used innovative approaches in this study, the results should be interpreted considering the limitations. The deliberate displacement of DHS cluster location data reduces the precision of travel time estimates and could lead to type two error where we failed to observe an existing significant association between travel time and SBA. There might be errors in classifying women as having SBA or not, as women might not remember birth details well or know the exact designation of health workers attending to them [43]. The reference period for EmONC survey signal function questions is three months, but the data used are ten years old. Therefore, service provision could have changed over the years.

We assumed that women who used SBA attended the nearest health facility due to insufficient location data in the DHS, but we know that a large proportion of lower tier health facilities with lower quality might be bypassed and underutilised [44, 45]. Therefore, although travel time to health facilities on average is short, women might travel longer distances or referred to hospitals for lifesaving services such as surgery and blood transfusion. Thus, we could be underestimating the impact of quality on SBA. Future studies can avoid this challenge by using HMIS data that names the community, health facility used and referral data enabling true catchments and health districts to be captured [44]. Also, poorer women living near private health facilities in urban areas might not be able to afford care at that nearest health facility.

Our travel impedance measure is more realistic compared with Euclidean and network distance [46]. However, travel speeds could vary by land cover type [28], and where detailed digitised roads are available, tracks and trails could be assigned different speeds [47].

The quality of care in health facilities could be misclassified due to outdated SPA data as there is an eight-year lag between the DHS survey and the SPA. The quality of care provided in the health facilities might have improved, deteriorated or unchanged within this period. Kaselitz and colleagues, for instance, found that a health facility improved in the Upper East region [48].

Lastly, we weighted the proportions but not the multilevel model although the DHS is a complex survey. Regardless, the inferences from a weighted versus unweighted multilevel model are often similar [49].

There was not much difference in the average travel time to health facilities in Ghana compared to other African countries such as Kenya, Malawi, Nigeria and Tanzania [50]. However, design of the health system and policies in Ghana implies findings in this study should be cautiously generalised. There are free maternal care and unrestricted access to secondary health facilities in Ghana, which is not the case in many other African countries [51]. Also, SBA has increased and travel time to health facilities providing maternal care has been reduced substantially due to the rapid increase in primary care facilities through the CHPS initiative [52].

## Conclusion

After controlling for established individual-level determinants of SBA uptake, we found that higher service quality at nearby healthcare facilities increased SBA, whereas travel time to nearest facility was unrelated to SBA. This suggests that following initiatives to expand the network of primary healthcare facilities in Ghana, a drive to improve service quality at these facilities could further widen SBA uptake. Our study also highlights the value of spatial linkage of household survey and health facility databases. In future work, we recommend examining services at healthcare facilities from the patient perspective in addition to using clinical SPA, to account for unexplained geographic variation in SBA. Similar approaches integrating different types of data can be applied to other health problems in Ghana and elsewhere.

## Supplementary Material

Supplementary Information

## Figures and Tables

**Table 1:** Bivariate association between background characteristics and skilled attendance at birth. Frequencies and percentages are weighted (weighted *N* = 11106, unweighted *N* = 11656)

		No SBA (%)	SBA (%)	Chi-square	Total for all categories (%)
Education	No formal education	951 (36.6)	1647 (63.4)	846 (*P* < 0.001)	2598 (25.4)
	Primary	1056 (17.0)	5166 (83.0)		6222 (56.0)
	Secondary	102 (6.5)	1479 (93.5)		1581 (14.2)
	Tertiary	8 (1.1)	697 (98.9)		705 (6.3)
Health Insurance	No	424 (33.6)	839 (66.4)	194 (*P* < 0.001)	1263 (11.4)
	Yes	1693 (17.2)	8150 (82.8)		9843 (88.6)
Age group	15–20	173 (19.0)	739 (81.0)	3.97 (0.265)	912 (8.2)
	20–30	917 (18.7)	3987 (81.3)		4904 (44.2)
	30–40	819 (18.9)	3504 (81.1)		4323 (38.9)
	40–50	207 (21.4)	759 (78.6)		966 (8.7)
ANC	1–3	419 (46.6)	480 (53.4)	481 (*P* < 0.001)	899 (8.1)
	4 or more	1698 (16.6)	8509 (83.4)		10207 (91.9)
Location	Rural	1644 (29.3)	3959 (70.7)	774 (*P* < 0.001)	5603 (50.5)
	Urban	473 (8.6)	5030 (91.4)		5503 (49.5)
Parity	1–2	666 (13.1)	4433 (86.6)	395 (*P* < 0.001)	5099 (46.4)
	3–4	634 (18.4)	2815 (81.6)		3449 (31.1)
	5 or more	817 (32.0)	1740 (68.0)		2557 (22.5)
Wealth	Poor	1562 (33.9)	3051 (66.1)	1224 (*P* < 0.001)	4617 (41.5)
	Middle	345 (15.4)	1892 (84.6)		2237 (20.1)
	Rich	210 (4.9)	4045 (95.1)		4255 (38.3)
Median travel time to the nearest health facility	Within one hour	1986 (18.4)	8803 (81.6)	109 (*P* < 0.001)	10789 (97.1)
	One to two hours	107 (43.7)	138 (56.3)		245 (2.2)
	Two or more hours	24 (33.3)	48 (66.7)		72 (0.6)
Median travel time to the nearest health facility providing birthing services	Within one hour	1971 (18.4)	8756 (81.6)	146 (*P* < 0.001)	10727 (96.6)
	One to two hours	102 (40.2)	152 (59.8)		254 (2.3)
	Two or more hours	45 (35.7)	81 (64.3)		126 (1.1)
Median travel time to the nearest hospital/secondary facility	Within one hour	1665 (17.3)	7921 (82.7)	143 (*P* < 0.001)	9576 (86.2)
	One to two hours	344 (30.6)	781 (69.4)		1125 (10.1)
	Two or more hours	118 (29.1)	287 (70.9)		405 (3.6)
Highest EmONC facility near cluster	No EmONC data	613 (38.9)	962 (61.1)	707 (*P* < 0.001)	1572 (14.2)
	Non-EmONC	565 (25.5)	1655 (74.5)		2220 (20.0)
	Partial/Basic	611 (16.4)	3133 (83.6)		3724 (33.5)
	Comprehensive	328 (9.1)	3258 (90.9)		3586 (32.3)

**Table 2:** Multilevel model results predicting the use of SBA using travel time to the nearest health facility, nearest health facility providing birthing services and the nearest hospital

Effects		Travel to the nearest health facility	Travel to nearest health facility providing birthing services	Travel to the nearest hospital providing birthing service
Fixed effects		Odds ratio (95% CI)	Odds ratio (95% CI)	Odds ratio (95% CI)
Personal level factors				
Education	No formal education	1	1	1
	Primary	1.63 (1.42, 1.87) ***	1.63 (1.42, 1.87) ***	1.63 (1.42, 1.87) ***
	Secondary	2.78 (2.15, 3.59) ***	2.78 (2.15, 3.6) ***	2.78 (2.15, 3.59) ***
	Tertiary	11.79 (5.32, 26.16) ***	11.79 (5.31, 26.15) ***	11.8 (5.32, 26.17) ***
Health Insurance	No	1	1	1
	Yes	1.69 (1.43, 1.99) ***	1.69 (1.43, 1.99) ***	1.69 (1.43, 1.99) ***
ANC	1–3	1	1	
	4 or more	2.9 (2.44, 3.44) ***	2.9 (2.44, 3.44) ***	2.9 (2.44, 3.44) ***
Parity	1–2	1	1	1
	3–4	0.77 (0.67, 0.88) ***	0.77 (0.67, 0.88) ***	0.77 (0.67, 0.88) ***
	5 or more	0.6 (0.52, 0.69) ***	0.6 (0.52, 0.69) ***	0.6 (0.52, 0.69) ***
Wealth	Poor	1	1	1
	Middle	1.61 (1.35, 1.93) ***	1.61 (1.35, 1.93) ***	1.61 (1.35, 1.93) ***
	Rich	2.86 (2.29, 3.58) ***	2.86 (2.29, 3.57) ***	2.88 (2.31, 3.59) ***
Cluster-level factors				
Location	Rural	1	1	1
	Urban	2.06 (1.67, 2.53) ***	2.05 (1.66, 2.52) ***	2.06 (1.67, 2.54) ***
Highest EmONC facility near a cluster	No EmONC data	1	1	1
	Non-EmONC	1.38 (1.05, 1.82) *	1.38 (1.05, 1.81) *	1.4 (1.07, 1.85) *
	Partial/Basic	2.02 (1.53, 2.66) ***	2.01 (1.52, 2.65) ***	2.05 (1.55, 2.72) ***
	Comprehensive	2.28 (1.68, 3.09) ***	2.28 (1.68, 3.08) ***	2.31 (1.7, 3.14) ***
Travel time (hours)	Travel time (hours)	0.87 (0.69, 1.11)	0.85 (0.67, 1.08)	1 (0.87, 1.16)

Effect is significant at ***P < 0.001, *P < 0.05.

**Table 3:** Effect of cluster and region on SBA

	Travel to the nearest health facility	Travel to nearest health facility providing birthing services	Travel to the nearest hospital providing birthing service
Random effects	Variance (SD)	Variance (SD)	Variance (SD)
Cluster	0.71 (0.84)	0.71 (0.84)	0.72 (0.85)
Region	0.31 (0.56)	0.31 (0.55)	0.31 (0.56)

SD, Standard deviation.
