# Supplementary figures and images for: Spatial inequalities in skilled attendance at birth in Ghana: a multilevel analysis integrating health facility databases with household survey data
Source: Trop Med Int Health. Author manuscript; Available in PMC 2022 Sep 6. (PMC7613541; doi:10.1111/tmi.13460)

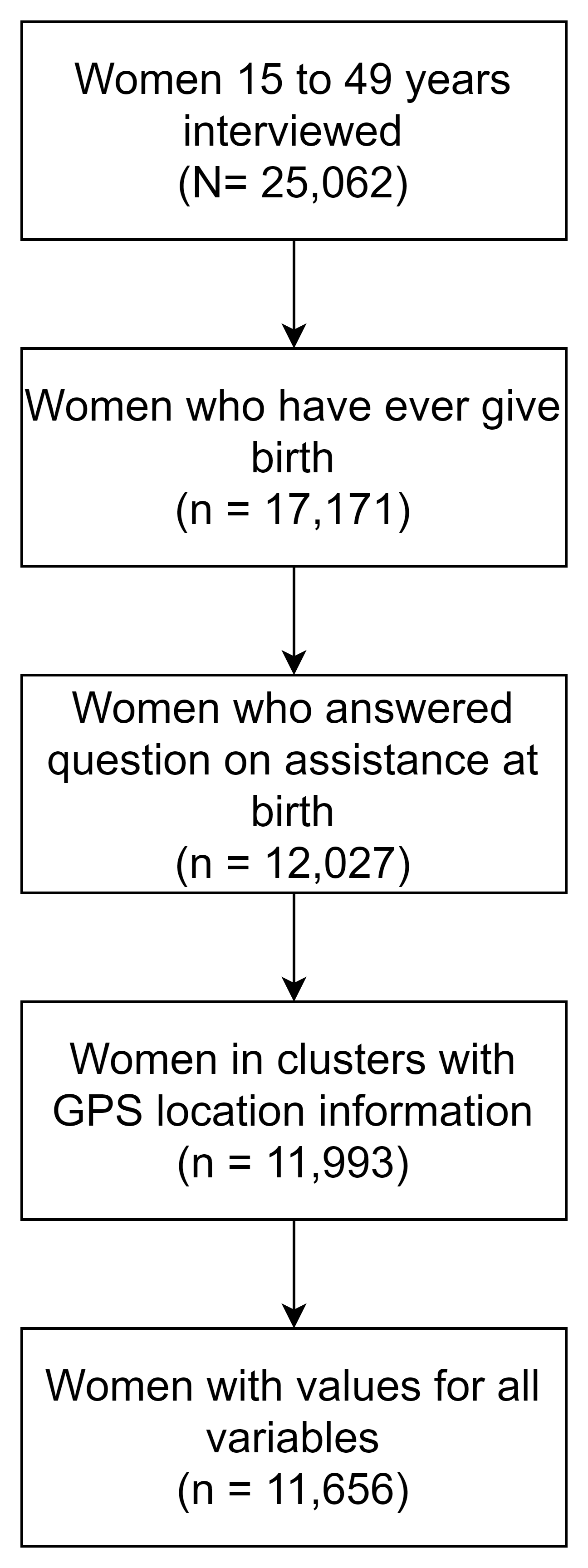

Supplement: Supplementary Information [file EMS153514-supplement-Supplementary_Information.zip › tmi13460-sup-0001-figs1.tif]

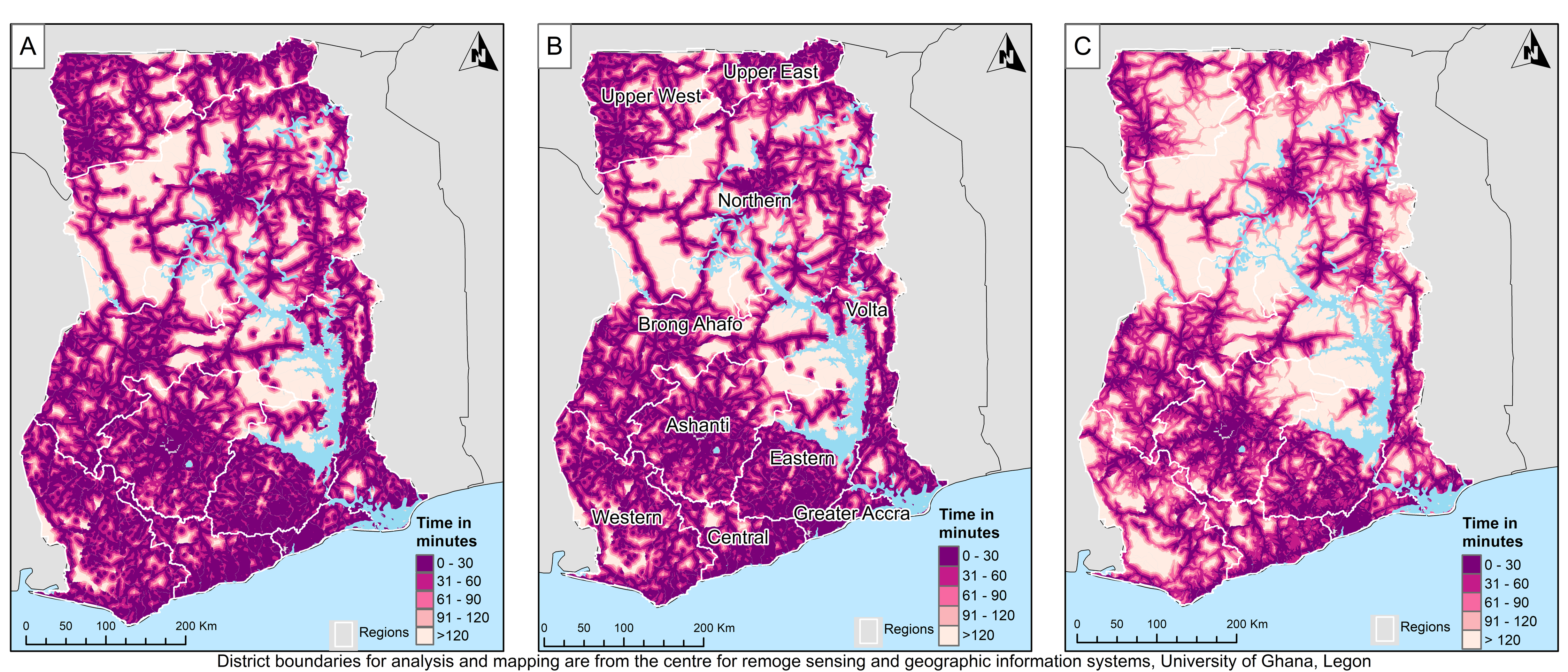

Supplement: Supplementary Information [file EMS153514-supplement-Supplementary_Information.zip › tmi13460-sup-0002-figs2.tif]
